# Topical Treatment of Nonhealing Venous Leg Ulcer with Propolis Ointment

**DOI:** 10.1155/2013/254017

**Published:** 2013-04-14

**Authors:** M. Kucharzewski, M. Kózka, T. Urbanek

**Affiliations:** ^1^Department of Descriptive and Topographic Anatomy, Medical University of Silesia, ul. Jordana 19, 41-808 Zabrze, Poland; ^2^Outpatient Surgery Center no. 2, Specialist Hospital no. 2, ul. Batorego 15, 41-902 Bytom, Poland; ^3^5th Military Hospital, ul. Wrocławska 1-3, 30-901 Cracow, Poland; ^4^Department of General and Vascular Surgery, Medical University of Silesia, ul. Ziołowa 45/47, 40-635 Katowice, Poland

## Abstract

An investigation of effectiveness of topical treatment of nonhealing chronic venous leg ulcers with propolis ointment was conducted. 56 patients were included in the study and randomized into two groups. In group 1, there were 28 patients (ulceration area: 6.9–9.78 cm^2^) treated by means of topical propolis ointment application and short stretch bandage compression. In group 2, there were 29 patients (ulceration area: 7.2–9.4 cm^2^) treated by means of Unna boot leg compression without topical propolis treatment. In the study, the efficacy of both treatment methods in patients with resistive venous leg ulcers was compared. The ulceration of patients from group 1 healed completely after 6 weeks of therapy in all cases. In all patients from group 2, the process of healing was longer but successfully completed after 16 weeks of the therapy. We found that an adjunctive propolis ointment treatment increases the efficacy of the short stretch bandage compression stocking, and this combined treatment is more effective than Unna's boot compression alone.

## 1. Introduction

Venous leg ulcer represents a significant medical problem as well as economic and social burden. Many ulcers are chronic and reveal poor responses to usual therapies. Baker et al. [1] reported that resistive ulcers persisting for more than one year concern 24% of patients with chronic crural ulcer of the venous origin. 35% of patients had ulceration problem for more than five years, and 20% had suffered 10 or more episodes of ulceration. The occurrence and the treatment of chronic leg ulcer generate significant costs related to the necessity of often expensive and long-term therapy [1]. In the United States alone, between 500,000 and 1,000,000 people suffer from chronic leg ulcers, the majority of which result from venous insufficiency [2]. An improvement in the knowledge concerning venous ulcer pathophysiology as well as an implementation of modern compression therapy and TIME strategy in chronic wound treatment significantly improved the rate of the healed ulcers. However, despite this progress, the number of patients with resistive or recurrent venous ulcer still remains relatively high. This fact stimulates new studies as well as an implementation of new methods and medicines in this field [3, 4].

Propolis is a resinous substance collected from trees by the bee *Apis mellifera*, which uses it as a building and insulating material in the hive. Bees use propolis (bee glue) not only as a building material, but also to keep low concentration of bacteria and fungi in the hive. Although its chemical composition varies, propolis constituents generally include about 10% essential oils, 5% pollen, and 15% various organic polyphenolic compounds including flavonoids and phenolic acids. Propolis is remarkably used in dermatology for wounds healing, burn and external ulcers treatment, healing time reduction, wound contraction increase, and tissue repair acceleration. During wound healing, perfect synchronized cellular and molecular interactions occur to repair damaged tissue [5–7].

The main objective of this study is to investigate the use of propolis ointment in the treatment of chronic nonhealing venous leg ulcers.

## 2. Materials and Methods

### 2.1. Patients

Our research comprised 56 patients with chronic venous ulceration with a history of active crural ulcer ≥12 months and the lack of complete healing despite previous nonsurgical treatment. After clinical examination, the venous origin of the ulcer was confirmed by means of the venous duplex Doppler sonography and ABI measurement. The patients with previous or active deep vein thrombosis were excluded from the study. Additional exclusion criteria were chronic or critical leg ischemia, contraindications to compression therapy, immobilization in orthesis or plastic cast, paresis related to stroke or paraplegia, chronic cardiac failure with peripheral swelling, systemic infection, and previous propolis implementation. In all cases according to the laboratory data, diabetes was also excluded. Patients treated pharmacologically with sulodexide or other anticoagulants as well as those on pentoxifylline or prostaglandins were not included in the study. 

 The consecutive patients qualified to the study were randomly divided into two groups. The randomization numbers were given to the patients according to the order of the inclusion of the consecutive patients into the study (group 1 included the patients with odd numbers and group 2 included those with even numbers). No patient suffered from clinically significant peripheral atherosclerotic occlusive disease; in all cases the ankle-brachial index was ≥0.8. 

Group 1 consisted of 17 women and 11 men aged 50–78 years (average 66.5 years). Their ulceration area varied from 6.9 cm^2^ to 9.78 cm^2^ (average 8.35 cm^2^). The ulcer location of 13 patients was on the left crus, and of 15 patients on the right one. The wound persisted at the beginning of therapy for 15–38 months (average 21 months). 22 patients were fully mobile; 6 women had limited mobility. Body mass index (BMI) ranged from 22.1 to 38.6 kg/cm^2^ (average 29.8 kg/cm^2^). It was >30 kg/cm^2^ in 3 women and two men (Table 1). 

Group 2 consisted of 18 women and 10 men aged between 52 and 70 years (average 60.6 years). The ulceration area varied from 7.2 to 9.4 cm^2^ (average 8.29 cm^2^) and the duration of the wound from 13 months to 38 months (average 23 months). In 13 patients, ulcers were located on the left crus, and in 15 patients on the right one. Full mobility was possible for 20 patients and a limited one for 8 patients. BMI ranged from 28.5 to 41.2 kg/cm^2^ (average 28.9 kg/cm^2^). It was >30 kg/cm^2^ in 6 women and 3 men (Table 1).

On the basis of clinical and sonographic results, the chronic venous disease of each patient was classified according to CEAP classification (Table 2). 

Each patient provided history of the index lesion, treatment, and other significant medical conditions. All patients had been previously treated by general practitioners by means of elastic bandage compression stocking with wound antiseptic lavage and local application of traditional dressing such as hydrogel and hydrocolloid dressing. However, none of these methods resulted in complete healing of the wound within prerandomization period.

 Each ulcer was classified according to wound morphology, severity, and location. We recorded a systematic description of wound and limb appearance, including edema, erythema, exudation, granulation, and presence of fibrin or eschar. 

### 2.2. Methods

The ulcers were thoroughly debrided with removal of fibrin, eschar, and, if present, necrotic tissue. The necrotic tissues had been removed from ulcerations by surgery or with the aid of enzyme-containing ointment and antiseptic lavage.

For the local treatment in group 1, topical application of 7% propolis ointment (Farmina, Cracow, Poland) was used. The ulcers were rinsed with physiological sodium chloride solution, and then the propolis ointment was applied. Then gauze pads were placed on the ulcer, and the limb was bandaged with non-elastic supportive bandage. The compression short-stretch bandages were used for all the patients in this group. A spiral two-layer bandaging technique with the 10 cm wide bandage applied from toe to knee was used for the compression. The propolis ointment was changed every day until the ulcer was healed. For the proper compression pressure measurement, the Kikuhime pressure sensor was used and the pressure of 25–35 mm Hg was applied.

Patients from group 2 underwent the compression treatment by means of Unna's boot. After rinsing the wound with physiological sodium chloride solution, Unna's rigid paste bandage was tied around the limbs from below the toes up to the knee. This dressing was changed every 7 days until the ulcer was healed. 

Prior to treatment, a bacterial swab was taken from each ulcer. During the wound dressing and compression changes, the area of the ulcers was always evaluated. The procedure was as follows. Firstly, homothetic congruent projections of the ulcers were plotted onto transparent foil, after which planimetric measurements of the wounds were taken with the use of digitizer Mutoh Kurta XGT-1218A3 (USA). The area of the ulcer was determined once a week until the wound healed completely. The speed with which the ulceration area was decreasing daily was calculated for each patient according to the formula

(1) $$\begin{array}{c} v_{s}=\frac{S_{i-1}-S_{i}}{t_{}}, \end{array}$$

where *S*_*i*_ is the ulceration area at the time of the previous measurement; *S*_*i*−1_ is the ulceration area on the day of a given measurement; *t* is the time (in days) in which the ulceration area was changing from *S*_*i*−1_ to *S*_*i*_; *i*: 1, 2—number of the group.

All patients underwent ambulatory treatment. They were examined by the physician every week, until the ulceration healed completely. The drug therapy in both groups followed a standard regimen. All patients received micronized flavonoid fraction (450 mg diosmin, 50 mg hesperidin), 2 tablets of 500 mg once daily.

 The results were analyzed by using Mann-Whitney *U *test; *P* values <0.05 were considered as statistically significant.

## 3. Results

In all 28 patients of group 1, the ulcers were healed successfully within the first 6 weeks of the treatment by means of two-layer short stretch bandage and topical propolis ointment application. The complete ulcer healing was also observed in all cases of group 2 (Unna boots without topical propolis application); however, within the first 6 weeks of treatment, the complete healing was found only in 6 out of 28 patients in this group (the time up till the healing of the last case in group 2 was significantly longer—16 weeks) (Table 3).

The mean time of the ulcer healing in group 1 was significantly shorter (24 days) than in group 2 (76 days) (*P* < 0.005). 

In group 1, after 7 days of treatment, the average ulceration area diminished by 1.93 cm^2^ and the speed of decreasing of the wound formation (*v*_1_) was 0.28 cm^2^/d. After another 7 days, the speed with which the ulcer area was decreasing equal to 0.20 cm^2^/d. The average area of the wound decreased to 1.42 cm^2^. The ulcers in three patients were healed. After the following 14 days of using compression and the propolis ointment, the ulcers of five women and four men healed completely; the average wound area of the remaining patients decreased to 1.98 cm^2^. After further seven days, the ulcers of five more patients had healed at the speed of *v*_1_ = 0.18 cm^2^/d. The average area of the wound decreased to 0.72 cm^2^. The healing of the last three patients' ulcers was completed after the next seven days of treatment. The speed of decreasing of the wound area in that period was *v*_1_ = 0.10 cm^2^/d.

Group 2 consisted of 28 patients with the average ulceration area of 8.29 cm^2^. After seven days of treatment, the average wound area decreased by 1.42 cm^2^, and the speed at which it decreased was 0.20 cm^2^/d. After further 7 days, the speed of decreasing of the ulceration area was equal to 0.13 cm^2^/d. The average wound area was 5.94 cm^2^. After the following 14 days of treatment, the average ulcer area was 5.10 cm^2^. It was diminishing at the speed of *v*_2_ = 0.08 cm^2^/d. On the 42th day of treatment, the healing of wound was complete in six women. The average ulcer area of the other patients was increased (5.19 cm^2^), and the speed at which it increased was *v*_2_ = −0.01 cm^2^/d. After further 7 days, the speed of the increase of the ulceration area was equal to 0.007 cm^2^/d. The average wound area was 5.24 cm^2^. After seven days of treatment, the average wound area decreased by 0.03 cm^2^ and the speed at which it decreased was 0.004 cm^2^/d. After the next 21 days of treatment, further ulcers were healed in five patients, and the average wound area in the other cases was equal to 4.02 cm^2^ with *v*_2_ = 0.15 cm^2^/d. After further 14 days, the speed of the increase of the ulceration area was equal to 0.11 cm^2^/d. The average wound area was 1.54 cm^2^. In five patients, the ulcers were healed. After the following 14 days of treatment, the average ulcer area was 0.74 cm^2^. It was diminishing at the speed of *v*_2_ = 0.06 cm^2^/d. The ulcerations of the remaining patients in this group healed after further 14 days of treatment, and the speed at which the ulceration area decreased was equal to *v*_2_ = 0.03 cm^2^/d. The mean ulceration area in groups 1 and 2 in the course of the treatment is presented in Table 4. 

In the group of patients treated with compression and propolis no adverse event related to the propolis, application was observed. In the course of the study, none of the patients rejected the proposed treatment in both treatment groups. 

## 4. Discussion

Venous leg ulcer is one of the biggest clinical problems in phlebology. Despite epidemiological and pathophysiological knowledge improvement, the number of patients suffering from this complication still remains high stimulating the research focused on more effective treatment methods. According to the previously performed studies as well as according to the daily clinical practice, the compression therapy is crucial for the healing of the venous leg ulcers, although the local therapy may also improve the healing rate if correctly applied to the wound. In this respect the crucial role of the TIME strategy and proper wound dressing should also be emphasized including many currently available nonocclusive or occlusive dressings such as hydrogels, hydrocolloids, alginates, or foams. In our investigation, in one of the study groups, we used the propolis ointment combined with double-layer compression stocking [3, 4]. 

The wound healing process involves many complex factors. These may be classified as local factors, systemic factors, and organ as well as species variability in response to injury. Topical medications should provide a specific desired effect during the appropriate stage of healing.

The propolis skin cream appears to have beneficial effects on burn wounds, inflammation of the skin, and other skin lesions [8–13]. According to the results of our study, it can also be concluded that propolis skin ointment appears to have beneficial effects on healing venous ulcers. Propolis has been shown to stimulate various enzyme systems, cell metabolism, circulation, and collagen formation as well as improve the healing of burn wounds [14]. In the study published by Kurson [15], sixty-four patients with tibial skin ulcers, aged from 23 to 98 years old, were treated using propolis extract in an ointment form. The ointment was applied daily to the ulcerated area, which was also treated peripherally with antibiotic ointments. The treatment lasted for 4–12 weeks. At the end of the treatment, 19 out of the 84 treated patients displayed no clinical signs of the condition, but 19 exhibited improved condition [15]. 

Another study of topical application of propolis on wounds, burns, and ulcers showed up to an 80% accelerated healing process compared to the controls using routine healing regimes [16]. Treated individuals (229 patients in total) underwent applications of the propolis containing cream at two propolis concentrations (2% and 8%). The higher concentration caused local intolerance in 18% of patients by day 9, whereas the lower concentration caused adverse symptoms in only 1.8% of patients by day 16. Burns and wounds treated with the low concentration cream healed after 11 days on average and septic wounds after 17.5 days and 67% of ulcers were healed in 38 days [16]. In our study, we did not observe any adverse events related to the 7% propolis ointment application, and similarly to the above-mentioned studies, an acceleration of the healing process was observed. In order to evaluate the results of the treatment,an assessment of the daily decrease of the ulceration area in mm^2^ or the percentage of the initial area was performed and compared between the groups. The speed of the decrease of the ulcer area in group 1 varied from 0.28 to 0.10 cm^2^/d whereas in group 2 it varied from 0.20 to 0.08 cm^2^/d. The comparison of the results from both groups showed that *v*_*s*_ of patients from group 1 was significantly higher than *v*_*s*_ of patients from group 2. The ulceration area of the patients included in our study was comparable between the group (group 1: 8.35 cm^2^; group 2: 8.29 cm^2^). All ulcerations of the patients who received treatment with compression stocking and propolis ointment healed much earlier than wounds of the patients in group 2 (treated with Unna boots only).

Various forms of compression therapy have been applied over the years. At present there is a wide variation in the management of venous leg ulcers. In USA, Unna's boots (a noncompliant, plaster-type bandage) are often applied in many crural ulcer patients; in UK, multilayer elastic compression is widely used, while in mainland Europe and Australia, the inelastic, short stretch bandaging is commonly prescribed [3, 17, 18]. According to the recent clinical evidence analysis published in 2011 in the venous ulcer healing, compression bandages and stockings cure more ulcers compared with no compression, but there is still very little evidence-supported data answering the question: which bandaging technique is most effective [4]? There is also no high-quality evidence comparing the efficacy of multilayer high compression bandages, short stretch bandages, or Unna's boot nonelastic system compression. In our study, two systems were used including short stretch bandages, and Unna's boots, and in all the cases the compression was applied under physician's supervision (with local pressure measurement). In the group of combined treatment (short stretch bandages and propolis), the velocity of venous ulcer healing was significantly higher than in the group of non-elastic Unna's boot compression. 

In patients treated with an implementation of the non-elastic compression (Group 2), the aspect of the proper patient mobility should also be evaluated. We did not observe statistically significant differences concerning the rate of patients with limited mobility between the groups however, in the Unna's boot treated patients, one-third of the patients (10 out of 28 cases) represented the group with restricted mobility (in the group, in 6 out of 28; *P* > 0.05). All the patients qualified to the study underwent previous (but unsuccessful) compression treatment (considered by their general practitioners). In the study, the compression was applied and controlled by the study physicians to ensure the proper implementation of this therapeutic tool. The results achieved in the study emphasized the role of proper compression as well as proper patient compliance to the advised compression stocking or bandages. In both groups, in all the patients ulceration was cured; however, in the group supported by the propolis ointment application, the time to the complete ulcer healing was shorter. 

Despite the encouraging venous ulcer treatment efficacy achieved in our study protocol, further studies concerning propolis implementation in this clinical situation should be projected. In this setting, the necessity of further randomized controlled trial with head to head comparison of the same compression treatment with and without local propolis applications should be emphasized. The results of the performed study encourage us to plan such a trial in the very near future. 

## 5. Conclusion

We found that an adjunctive propolis ointment treatment increases the efficacy of the short stretch bandage compression stocking and this combined treatment is more effective than Unna's boot compression alone.

## Figures and Tables

**Table 1:** Characteristics of the groups.

	Group 1	Group 2	Statistical significance
Number of patients	28	28	n/s
Women	17	18	n/s
Men	11	10	n/s
Age (years)	50–78	52–70	n/s
Ulcer location—crural medial	18	17	n/s
Ulcer location—crural lateral	10	11	n/s
Ulcer area at the beginning (cm^2^)	6.9–9.78	7.2–9.4	n/s
Duration of ulceration (months)	15–36	13–38	n/s
Full mobility	22	18	n/s
Limited mobility	6	10	n/s
Ankle-brachial pressure index	0.92–1.12	0.97–1.15	n/s
Body mass index (kg/cm^2^)	22.1–38.6	28.5–41.2	n/s

**Table 2:** Classification of venous dysfunction (according to the CEAP classification).

CEAP class	Group 1	Group 2
C_6_E_P_A_S2,3_P_R_	9	10
C_6_E_P_A_S3D18_P_R_	7	8
C_6_E_P_A_S3D18P18_P_R_	6	5
C_6_E_P_A_S4_P_R_	6	5

**Table 3:** Patients with healed ulcers according to the duration of treatment.

Group	Duration of treatment (weeks)															
	1	2	3	4	5	6	7	8	9	10	11	12	13	14	15	16
1	4	4	8	4	5	3										

2				6				5				5		5	4	3

**Table 4:** Mean ulceration area (cm^2^) according to the duration of treatment.

Duration of treatment	Group 1		Group 2		Statistical significance between groups
	Ulceration area	*N*	Ulceration area	*N*
Before	8.35 ± 0.72^a^	28	8.29 ± 0.80^a^	28	NS
1 week	6.42 ± 0.82^a^	24	6.87 ± 0.69^a^	28	NS
2 weeks	5.00 ± 0.89^a^	20	5.94 ± 0.73^a^	28	*P* < 0.001
3 weeks	3.44 ± 0.72^a^	12	5.36 ± 0.73^a^	28	*P* < 0.001
4 weeks	1.98 ± 0.88^a^	8	5.10 ± 0.76	22	*P* < 0.001
5 weeks	0.72 ± 0.22^a^	3	5.19 ± 0.81	22	*P* < 0.001
6 weeks	0.00	0	5.24 ± 0.88	22	
7 weeks			5.21 ± 1.06^a^	22	
8 weeks			4.61 ± 1.2^a^	17	
9 weeks			4.02 ± 1.22^a^	17	
10 weeks			3.11 ± 1.12^a^	17	
11 weeks			2.24 ± 1.09^a^	17	
12 weeks			1.54 ± 0.89^a^	12	
13 weeks			0.98 ± 0.49	12	
14 weeks			0.74 ± 0.30^a^	7	
15 weeks			0.38 ± 0.06^a^	3	
16 weeks			0.00	0	

Statistical significance ^a^ *P* < 0.005 compared with the next week following day of measurement.
